# Non‐glycosidic compounds can stimulate both human and mouse *i*NKT cells

**DOI:** 10.1002/eji.201546114

**Published:** 2016-03-01

**Authors:** John‐Paul Jukes, Uzi Gileadi, Hemza Ghadbane, Ting‐Fong Yu, Dawn Shepherd, Liam R. Cox, Gurdyal S. Besra, Vincenzo Cerundolo

**Affiliations:** ^1^ MRC Human Immunology Unit, Radcliffe Department of Medicine, MRC Weatherall Institute of Molecular MedicineUniversity of OxfordOxfordUK; ^2^ School of Biosciences, University of BirminghamEdgbastonBirminghamUK; ^3^ School of ChemistryUniversity of BirminghamEdgbastonBirminghamUK

**Keywords:** α‐Galactosylceramide, CD1d, Immunotherapy, *i*NKT cell, Tumor

## Abstract

Invariant natural killer T (*i*NKT) cells recognize CD1d/glycolipid complexes and upon activation with synthetic agonists display immunostimulatory properties. We have previously described that the non‐glycosidic CD1d‐binding lipid, threitolceramide (ThrCer) activates murine and human *i*NKT cells. Here, we show that incorporating the headgroup of ThrCer into a conformationally more restricted 6‐ or 7‐membered ring results in significantly more potent non‐glycosidic analogs. In particular, ThrCer 6 was found to promote strong anti‐tumor responses and to induce a more prolonged stimulation of *i*NKT cells than does the canonical α‐galactosylceramide (α‐GalCer), achieving an enhanced T‐cell response at lower concentrations compared with α‐GalCer both in vitro, using human iNKT‐cell lines and in vivo, using C57BL/6 mice. Collectively, these studies describe novel non‐glycosidic ThrCer‐based analogs that have improved potency in *i*NKT‐cell activation compared with that of α‐GalCer, and are clinically relevant *i*NKT‐cell agonists.

## Introduction

Invariant natural killer T (*i*NKT) cells are a specialized subset of T cells that recognize glycolipids presented in the context of the non‐polymorphic MHC‐class I like molecule, CD1d. The archetypical *i*NKT‐cell agonist is α‐galactosylceramide (α‐GalCer), although a large number of exogenous and endogenous CD1d agonists have now been described 1, 2, 3, 4, 5, 6, 7, 8, 9, 10, 11, 12, 13, 14, 15. *i*NKT cells occupy an important immunological niche and can be activated via a number of well‐described pathways. Signals received through the invariant T‐cell receptor (*i*TCR) following lipid–CD1d complex recognition are integrated with signals derived from cytokines within the microenvironment, from both the innate and adaptive arms of the immune system 16, 17, 18, 19, 20. Understanding the sequence of events following *i*NKT‐cell activation has demonstrated that these cells aid in the activation of dendritic cells (DC), natural killer (NK) cells, B cells, and enhance antigen‐specific cytotoxic T lymphocyte (CTL) responses 21, 22, 23, 24, 25, 26, 27. Conversely, *i*NKT cells have been shown to promote immunity through their capacity to suppress myeloid‐derived suppressor cells 28, 29. Since these properties are a prerequisite for successful vaccine adjuvants, *i*NKT‐cell agonists have emerged as attractive candidates for augmenting current vaccination strategies 30.

We, and others, have demonstrated that co‐administration of α‐GalCer with antigenic proteins, such as ovalbumin (OVA), is able to significantly enhance antigen‐specific T‐ and B‐cell responses 10, 26, 27, 31, 32. Importantly, we were able to show that *i*NKT‐cell activation can enhance priming of T cells specific to the human tumor antigen, NY‐ESO‐1 32, which is expressed in a broad range of human tumors.

Previous studies have shown that in vivo the pharmacokinetics properties of any given *i*NKT agonist can play a significant role in determining the overall potency of the compound, beyond its affinity to *i*NKT‐cell TCR 9, 13. Analogs of α‐GalCer with changes introduced in proximity to the polar headgroup persisted for longer on splenic CD11c^+^ cells compared with α‐GalCer and were able to stimulate for a longer period of time *i*NKT‐cell hybridomas. Therefore, analogs that can stimulate *i*NKT cells enough to provide significant adjuvant properties, yet contain a headgroup that provides resistance to rapid clearance, may be more desirable alternatives for clinical translation. In keeping with this reasoning, we have previously demonstrated the activity of the non‐glycosidic analog, threitolceramide (ThrCer) 33. In this study we focused our attention on strategies to increase the potency of ThrCer.

Here, we report on structural modifications made to ThrCer, designed to restrict flexibility in the sugar headgroup, specifically by incorporating the threitol unit into a carbocycle of varying sizes. We analyzed the human and murine *i*NKT‐cell TCR affinities of lipid–CD1d molecules in complex with these compounds. We found that the conformationally more restricted six‐ and seven‐membered ring analogs, hereafter referred to as ThrCer 6 and ThrCer 7, respectively, induced increased iNKT‐cell activity compared with ThrCer and in some assays, ThrCer 6 induced even stronger activity than does α‐GalCer. Both ThrCer 6 and ThrCer 7 activated murine and human *i*NKT cells. Importantly, we show a longer bioavailability of ThrCer 6 than α‐GalCer, as defined by its ability to activate *i*NKT cells both in vitro and in vivo. Such properties of non‐glycosidic *i*NKT‐cell agonists make them important and potentially clinically promising compounds for future *i*NKT‐cell based therapy.

## Results

### Carbocyclic analogs of ThrCer enhance the human CD1d/lipid–iNKT‐cell TCR interaction

**Figure 1:**
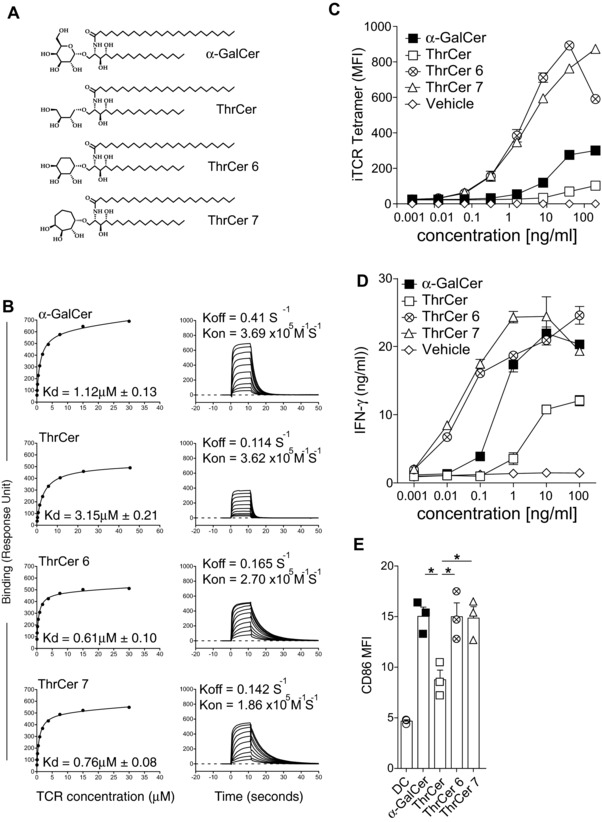
ThrCer 6 and ThrCer 7 are potent human *i*NKT‐cell agonists and display a prolonged interaction with the *i*NKT‐cell TCR when in complex with human CD1d molecules. (A) The structures of α‐GalCer and non‐glycosidic analogs ThrCer, ThrCer 6, and ThrCer 7 are shown. (B) Equilibrium binding and kinetics measurements of a soluble human *i*NKT‐cell TCR were assessed for hCD1d molecules refolded with α‐GalCer, ThrCer, ThrCer 6, and ThrCer 7. *K*_d_ values (μM) were calculated from equilibrium binding. (C and D) *i*NKT‐cell agonists were added to human CD1d C1R cells at various concentrations. After incubating overnight, the degree of *i*NKT‐cell recognition, assessed either (C) by using a tetrameric human *i*NKT‐cell TCR by flow cytometry or (D) by the addition of human *i*NKT cells to cultures and determining the amount of IFN‐γ released in supernatant by ELISA. (E) Human DC maturation was assessed following coculture with human *i*NKT cells and l μg lipids after 40 h, as determined by CD86 upregulation on DC by flow cytometry. Median fluorescent intensity = MFI. (D and E) Error bars are mean ± SEM (triplicate wells). Data shown are from single experiments representative of two experiments (B) or three experiments (C–E). *p < 0.05; Student's *t*‐test.

Two analogs of ThrCer, ThrCer 6, and ThrCer 7 were synthesized. Unlike ThrCer, these two analogs possess a conformationally more restricted six‐ or seven‐membered ring as the headgroup (Fig. 1A). We first carried out measurements to assess the binding affinity of these ThrCer analogs to human *i*NKT‐cell TCR (Fig. 1B). Recombinant human CD1d (hCD1d) molecules were refolded in vitro with ThrCer 6 and ThrCer 7 separately and coupled to BIAcore chips, over which refolded human *i*NKT‐cell TCR was injected at different concentrations 34. These experiments demonstrated that this specific human *i*NKT‐cell TCR displayed a significantly more prolonged interaction with hCD1d/ThrCer 6 and hCD1d/ThrCer 7, as compared with hCD1d/α–GalCer complexes. With both ThrCer analogs, a slower rate of dissociation (ThrCer 6: *k*_off_ = 0.165 s^−1^, ThrCer 7: *k*_off_ = 0.142 s^−1^, α‐GalCer: *k*_off_ = 0.41s^−1^) (Fig. 1B) resulted in higher affinity of binding of hCD1d/ThrCer 6 and hCD1d/ThrCer 7 complexes to the human *i*NKT‐cell TCR than that shown by hCD1d/α–GalCer complex (ThrCer 6: *K*_d_ = 0.61 ± 0.10 μM, ThrCer 7: *K*_d_ = 0.76 ± 0.08 μM, α‐GalCer: *K*_d_ = 1.12 ± 0.13 μM) (Fig. 1B). Importantly, these findings were consistent with data obtained using tetrameric human *i*NKT‐cell TCRs as a staining reagent to identify lipid/CD1d complexes on the surface of C1R cells that had been transfected with hCD1d cDNA (hCD1d C1R cells) and subsequently loaded with the different lipids 34 (Fig. 1C). The results of these experiments showed an enhanced staining by tetrameric human *i*NKT‐cell TCRs of hCD1d C1R cells upon incubation with ThrCer 6 and ThrCer 7 than with α‐GalCer (Fig. 1C).

To investigate whether these results could be confirmed and extended using polyclonal human *i*NKT‐cells, we next investigated the ability of ThrCer 6 and ThrCer 7 to activate human *i*NKT cells. To this end, hCD1d‐expressing C1R cells were loaded overnight with serial dilutions of lipids (α‐GalCer, ThrCer, ThrCer 6, and ThrCer 7) and then incubated with human *i*NKT cells. *i*NKT‐cell activation was assessed by measuring IFN‐γ production in the supernatant by ELISA. In keeping with the surface plasmon resonance (SPR) measurements, functional data confirmed a greater activation of human *i*NKT cells with ThrCer 6 and ThrCer 7 than with α‐GalCer, which was particularly evident at lower concentrations, below 1 ng/mL (Fig. 1D). These results were confirmed using a number of different human *i*NKT‐cell lines (data not shown). Furthermore, we observed an improved ability of ThrCer 6 and ThrCer 7 to induce human DC maturation compared to ThrCer, following presentation of lipids to *i*NKT cells, as defined by CD86 upregulation, which was comparable to that observed with α‐GalCer‐treated DCs (Fig. 1E). These results demonstrate that structural modifications in the headgroup of ThrCer, designed to restrict conformational flexibility of the headgroup lead to enhanced affinity of binding for human *i*NKT‐cell TCR and result in stronger human *i*NKT‐cell activation.

### Carbocyclic analogs of ThrCer enhance the murine CD1d/lipid–iNKT‐cell TCR interaction

**Figure 2:**
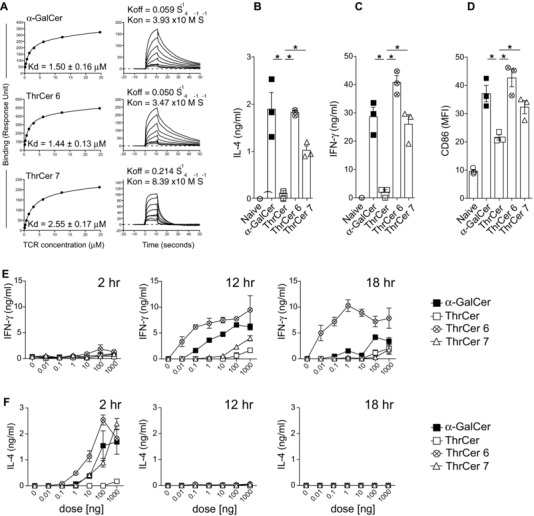
Enhanced in vivo potency of ThrCer 6. (A) Equilibrium binding and kinetics measurements of a soluble mouse *i*NKT‐cell TCR were assessed for mCD1d molecules refolded with α‐GalCer, ThrCer 6 and ThrCer 7. *K*_d_ values (μM) were calculated from equilibrium binding. (B–D) Mice were immunized with 1 μg lipids i.v. and (B) IL‐4 and (C) IFN‐γ were detected by ELISA in blood sera at either 2 or 18 h, respectively. (D) At 18 h, immunized mice were sacrificed and splenocytes stained with anti‐CD11c and anti‐CD86 antibodies to determine the extent of maturation by the expression of CD86 on gated DCs (CD11c^+^) using flow cytometry. (E and F) Mice were immunized i.v. with titrating doses of α‐GalCer, ThrCer, ThrCer 6, and ThrCer 7 and (E) IFN‐γ and (F) IL‐4 were detected in blood sera at 2, 12, and 18 h by ELISA. Median fluorescent intensity = MFI. (B–F) Data are shown as mean ± SEM (*n* = 3/group) and are representative of two independent experiments (A), three experiments (B–D), or a single experiment (E and F). *p < 0.05; Student's *t*‐test.

In order to extend the analysis of the ability of ThrCer analogs to activate *i*NKT‐cells in vivo, we assessed their ability to promote a response in a murine model. First, we performed binding assays using in vitro‐loaded murine recombinant CD1d (mCD1d) molecules and in vitro‐refolded murine Vα14/Vβ8 *i*NKT‐cell TCR. In contrast to the results obtained using human *i*NKT‐cell TCR, we observed that ThrCer 6 and α‐GalCer‐loaded mCD1d molecules had a prolonged interaction with the murine *i*NKT‐cell TCR and a similar affinity (α‐GalCer: *K*_d_ = 1.50 ± 0.16 μM; ThrCer 6: *K*_d_ = 1.44 ± 0.13 μM; Fig. 2A), while the rate of dissociation of the murine *i*NKT‐cell TCR from mCD1d/ThrCer 7 complex was faster, resulting in weaker binding (ThrCer 7: *K*_d_ = 2.55 ± 0.17 μM; Fig. 2A).

Consistent with the above findings, we were able to demonstrate a marked increase in the ability of ThrCer 6 to activate murine *i*NKT cells and mature DC in vivo, in a CD1d‐dependent manner (Supporting Information Fig. 1), which was significantly greater than ThrCer and similar to that of α‐GalCer (Fig. 2B–D). Interestingly, despite similar affinities of ThrCer 6 and α‐GalCer in the in vitro assays (Fig. 2A), in vivo titration of the dose of lipids demonstrated that intravenous (i.v.) injection of ThrCer 6 significantly enhanced IFN‐γ secretion, but not IL‐4 secretion, after 12 and 18 h, as compared with α‐GalCer (Fig. 2E and F). We extended these results by demonstrating that intramuscular (i.m.) injection of ThrCer 6 results, 18 h post i.m. injection, in higher IFN‐γ secretion as compared with α‐GalCer (Supporting Information Fig. 2). As a control, we demonstrated that the enhanced production of IFN‐γ in ThrCer 6 i.v. injected mice was not due to enhanced transactivation of NK cells (Supporting Information Fig. 3): harvesting splenocytes at either 12, 24, or 33 h post injection of ThrCer 6, ThrCer 7 and α‐GalCer and assessing the amount of intracellular IFN‐γ and surface expression of the activation marker CD69 on NK cells by flow cytometry showed comparable transactivation of NK cells for both ThrCer 6 and α‐GalCer (Supporting Information Fig. 3).

### ThrCer 6 and ThrCer 7 are potent iNKT‐cell agonists that can raise an immune response against tumors

**Figure 3:**
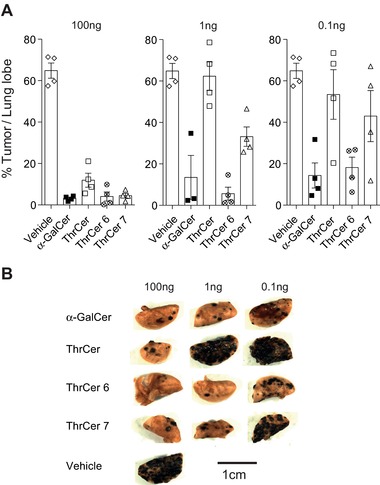
ThrCer 6 demonstrates robust anti‐tumor responses in a B16 melanoma tumor model. (A) Mice were immunized with 100, 1, or 0.1 ng of lipids i.v. 3 days before receiving an i.v. challenge with 5 × 10^5^ B16 melanoma cells (*n* = 4/group). Two weeks later the lungs were removed and the number of metastatic nodules quantified as described in section “Materials and methods”. Data in the three panels are shown as mean ± SEM of tumor area as percentage of total lung area, and are representative of two independent experiments. (B) Representative lung lobe from each group is shown. Photographic images are representative of four lung lobes in each of two independent experiments.

In order to demonstrate the efficacy of ThrCer 6 and ThrCer 7 as *i*NKT‐cell agonists, we tested their effects in a B16 melanoma model, in which *i*NKT cells play a pivotal role in initiating a NK‐cell and IFN‐γ response against tumor cells 35. To this end, mice were injected i.v. with a range of doses of lipids (100, 1, 0.1 ng) and 3 days later, challenged with i.v. injected 5 × 10^5^ B16 melanoma cells. Fourteen days later, the metastasis load in the lungs was quantified. Mice treated with the vehicle buffer had extensive tumor metastases (>70% of lung tissue), while all mice that had received 100 ng of lipids showed a marked reduction in the extent of tumor metastases (<10% of lung tissue; Fig. 3). With a reduction in lipid dose, the extent of tumor metastasis burden increased to varying degrees. Mice treated with α‐GalCer and ThrCer 6 showed far greater protection than did ThrCer‐treated mice and, even at a dose of a 1000‐fold lower than ThrCer, afforded comparable protection (Fig. 3).

**Figure 4:**
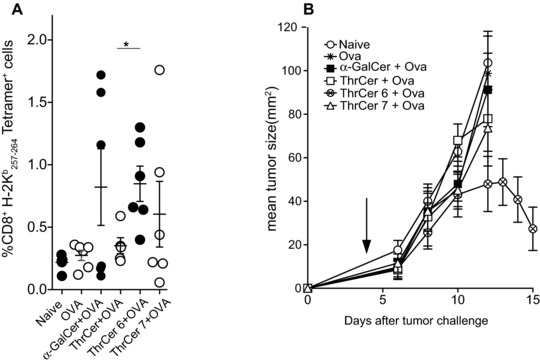
Injection of ThrCer 6 with OVA into tumor‐bearing mice results in tumor regression. Mice (*n* = 4–6) were injected s.c. with OVA‐expressing EL4 cells (EG7 cells). Four days later mice were injected i.v. with OVA protein together with either vehicle or 1 μg of the indicated iNKT‐cell agonist. (A) Seven days later mice were bled and the number of H‐2K^b^_257‐264_ tetramer^+^ cells was assessed by FACS analysis. (B) The size of the tumor was subsequently measured regularly using calipers and expressed as surface area. The arrow indicates the timing of the injection of soluble OVA plus *i*NKT‐cell agonists. (A and B) Data are shown as mean ± SEM and are representative of two independent experiments.

To test the functional consequences of *i*NKT‐cell‐induced DC maturation (Fig. 1E and Supporting Information Fig. 1) and to assess whether *i*NKT‐cell activation mediated by the injection of either ThrCer 6 or ThrCer 7 could lead to cross‐presentation of peptides derived from soluble antigenic proteins, mice were injected with the tumor cell line EL4 encoding the full‐length OVA and then 4 days later were immunized i.v. with full‐length OVA together with 1 μg of non‐glycosidic *i*NKT‐cell agonists. As a control, mice were injected with α‐GalCer with full‐length OVA. Seven days later, OVA‐specific CD8^+^ T‐cells were detected in the blood by H‐2 K^b^/OVA_257–264_ tetramer staining of CD8^+^ T cells. Injection of OVA together with ThrCer 6 and ThrCer 7 as well as α‐GalCer, resulted in a peptide‐specific response as seen by the number of T cells recognized by tetrameric MHC class I/peptide (Fig. 4A). Interestingly, under the conditions of these experiments, only mice that received ThrCer 6 with OVA rejected OVA‐expressing implanted tumors (Fig. 4B).

### ThrCer 6 displays enhanced activity compared with α‐GalCer at low concentrations

In both the human and mouse experimental models (Figs. 1D and 2E, respectively) treatment with ThrCer 6 produced a stronger IFN‐γ response than did treatment with α‐GalCer when using low concentrations of lipids (more so 18 than 12 h after stimulation (Fig. 2E)).

**Figure 5:**
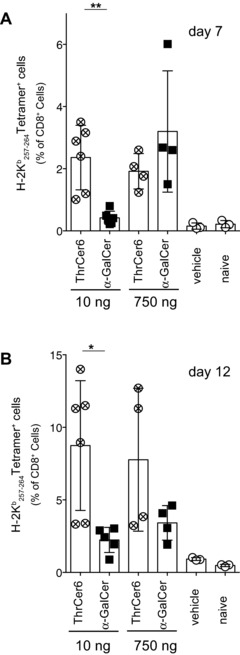
ThrCer 6 is a more potent immune adjuvant than α‐GalCer at low concentrations of lipid. (A and B) Mice were given 400 μg OVA together with i.v. injection of vehicle or 10 or 750 ng of either α‐GalCer or ThrCer 6. OVA‐specific CD8^+^ T cells were enumerated in the blood using tetrameric H‐2K^b^/OVA_257–264_ flow cytometry at (A) day 7 and at (B) day 12. Each square or circle represents the data for a single mouse (*n* = 4–6/group). Data shown as mean ± SEM and are representative of three independent experiments. **p = 0.0012, *p = 0.0114; Student's *t*‐test.

In order to assess the downstream effect of treatment with low concentrations of lipid in vivo, mice were injected with the model antigen OVA together with two different doses of ThrCer 6 and α‐GalCer. Seven days later, OVA‐specific CD8^+^ T cells were detected in the blood by H‐2 K^b^/OVA_257–264_ tetramer staining of CD8^+^ T cells. Injection of 750 ng of α‐GalCer or ThrCer 6 elicited a similar frequency of OVA‐specific CD8^+^ T cells (Fig. 5A). In contrast, injection of 10 ng of ThrCer 6 elicited a statistically significant higher frequency of OVA‐specific CD8^+^ T cells compared with mice injected with 10 ng of α‐GalCer (Fig. 5A). This higher frequency of OVA‐specific CD8^+^ T cells persisted at day 12 (Fig. 5B). Thus, cross‐presentation of peptides derived from OVA was markedly increased by concomitant stimulation of *i*NKT‐cell activity with ThrCer 6 compared to α‐GalCer.

**Figure 6:**
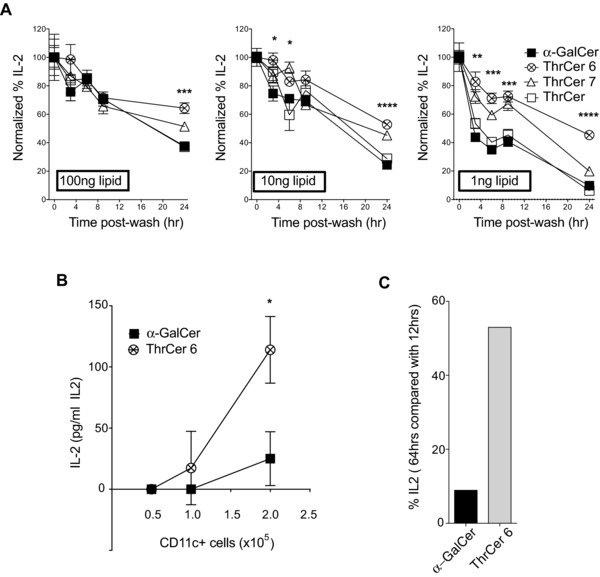
Enhanced bioavailability of ThrCer 6 as compared to ThrCer and α‐GalCer. (A) Mouse BMDCs were pulsed for 6 h with 100, 10, or 1 ng/mL of indicated *i*NKT‐cell agonists. Cells were washed and suspended in tissue culture medium in the absence of *i*NKT‐cell agonists for up to 24 h. Washed BMDCs were then cocultured with the murine *i*NKT cell hybridoma DN32.D3. Hundred percentage activation was defined for each *i*NKT‐cell agonist as the IL‐2 produced by DN32.D3 cocultured with BMDCs that were left with *i*NKT‐cell agonists throughout the pre coculture period (30 h). Diminished bioavailability is expressed as a percentage of the maximum DN32.D3 activation by each *i*NKT‐cell agonist. Number of replicates = 4. (B and C) Jα18 knockout mice were injected i.v. with 1 μg of ThrCer 6, α‐GalCer, or vehicle. Twelve hours or 64 h later, CD11c^+^‐purified cells were cocultured with 5 × 10^4^ DN32.D3 at different ratios. (B) IL‐2 in the supernatant from coculture of DN32.D3 with CD11c^+^ cells isolated after 64 h was determined by ELISA. (C) IL‐2 levels of the same culture as percentage of the IL‐2 produced by DN32.D3 coincubated with CD11c^+^ cells isolated 12 h after injection of lipids. *(*A and B) Data are shown as mean ± SEM and are representative of (A) three or (B and C) two independent experiments. Significance was calculated between α‐GalCer and ThrCer 6. *p < 0.05, **p < 0.01, ***p < 0.001, ****p < 0.0001; Student's *t*‐test.

The difference between the functional activity of ThrCer 6 and α‐GalCer, particularly noticeable at low concentrations, may be accounted for by differences in their bioavailability. To address this possibility, bone marrow (BM) derived DC were incubated with a range of different *i*NKT‐cell agonists at different concentrations overnight, then washed and incubated with fresh media for increasing lengths of time. Lipid‐pulsed DC were then used to stimulate the *i*NKT‐hybridoma DN32.D3 (Fig. 6A). At low concentrations of *i*NKT‐cell agonists (i.e. 10 and 1 ng), ThrCer 6 showed an enhanced ability to activate DN32.D3 compared with α‐GalCer, which was particularly evident when DC were pretreated with the *i*NKT‐cell agonists 24 h before coincubation with DN32.D3. The difference between the ThrCer 6 and ThrCer 7 analogs and α‐GalCer started to be evident as early as 3 h after lipid‐pulsed DC were washed (Fig. 6A).

To extend these results to an in vivo setting, purified CD11c^+^ cells, from ThrCer 6‐ or α‐GalCer‐injected mice, were used in vitro to activate the *i*NKT‐cell hybridoma DN32.D3. To rule out the possibility that the results of these experiments could be modulated by the in vivo killing of CD11c^+^ cells by *i*NKT cells, Jα18 knockout mice, lacking *i*NKT cells, were injected with ThrCer 6, α‐GalCer or vehicle, and then culled 12 or 64 h later. CD11c^+^ cells enriched from the spleens were then coincubated with DN32.D3 for 17 h and IL‐2 production was measured in the supernatant (Fig. 6B and C). Splenic CD11c^+^ cells from mice injected with ThrCer 6 were more potent activators of DN32.D3 cells 64 h after the in vivo injection compared with splenic CD11c^+^ cells from α‐GalCer‐injected mice (Fig. 6B). We showed that the ability of CD11c^+^ cells from α‐GalCer‐injected mice to stimulate DN32.D3 cells declined more rapidly than was the case with CD11c^+^ cells from ThrCer 6‐injected mice between 12 and 64 h post injection, as the decline in stimulation from α‐GalCer‐injected mice (at 64 h compared with 12 h) was more than 90% whereas the decline in stimulation by CD11c^+^ cells from ThrCer 6‐injected mice was less than 50% (Fig. 6C). These results provide convincing evidence that the in vivo bioavailability of ThrCer 6 is significantly longer than that of α‐GalCer.

## Discussion

We have described a novel series of non‐glycosidic *i*NKT‐cell agonists, which can promote strong anti‐tumor responses and induce a more prolonged stimulation of human and mouse *i*NKT cells than does the prototypical *i*NKT‐cell agonist, α‐GalCer.

*i*NKT cells occupy an important immunological niche at the intersection of the innate and adaptive immune responses. The use of pharmacological agents, such as α‐GalCer, capable of activating *i*NKT cells has led to an increasing interest in the use of *i*NKT‐cell agonists as immune adjuvants in the clinic. The clinical use of α‐GalCer in phase I trials for head and neck cancer demonstrated that such approaches are well tolerated in patients and provided evidence to support the development of *i*NKT‐cell agonists to enhance anti‐tumor responses, although additional clinical trials are still required (as reviewed in 36).

ThrCer was initially designed as a truncated form of α‐GalCer that maintains the OH groups corresponding to positions 2′, 3′, and 4′ OH of the galactose residue, which are needed for correct orientation of the headgroup in the CD1d/glycolipid complex to enable efficient recognition by the *i*NKT‐cell TCR 33, 37, 38, 39, 40. The acyclic nature of the threitol headgroup and its associated increased flexibility compared to that shown by the pyranose sugar in α‐GalCer, may be a factor contributing to the weaker ability of ThrCer to activate *i*NKT cells 33. In order to reduce the conformational flexibility of the threitol headgroup and in so doing hopefully increase the potential potency of analogs, we generated a series of compounds in which the threitol unit was embedded within a cyclitol ring (Fig. 1A). Our experiments demonstrated that this modification to the headgroup correlated to the observed enhanced binding affinities of the CD1d/lipid complexes to the *i*NKT‐cell TCR that were greater than that of CD1d monomers loaded with ThrCer (Figs. 1B and 2A). In murine studies, mCD1d/ThrCer 6 monomers had a *K*_d_ value similar to that of mCD1d/α‐GalCer monomers, with both compounds displaying a similar slow off‐rate (Fig. 2A). However, in human studies, both ThrCer 6 and ThrCer 7 displayed a more prolonged *i*NKT‐cell TCR engagement, resulting in *K*_d_ values greater than that shown by hCD1d/α‐GalCer monomers (Fig. 1B), thus identifying key differences between the engagement of murine and human *i*NKT‐cell TCR. Although the BIAcore measurements were done using refolded *i*NKT‐cell TCR isolated from a single clone of human and mouse *i*NKT cells, the functional data that supports the hierarchy of activity between the ThrCer analogs and α‐GalCer were obtained using polyclonal human *i*NKT cells and in‐vivo mouse studies.

The results from the binding affinity studies were further supported when we investigated the ability of ThrCer 6 and ThrCer 7 to activate murine *i*NKT cells, which confirmed the greater potency of ThrCer 6 and ThrCer 7 than ThrCer, and enhanced ability of ThrCer 6 to induce IFN‐γ secretion (Fig. 2E and Supporting Information Fig. 2) and elicit higher frequency antigen‐specific T‐cell responses than does α‐GalCer, particularly at lower doses (Fig. 5), while they had a similar effect on the number of B16 lung metastases. Importantly, injection of OVA in combination with ThrCer 6 in tumor‐bearing mice resulted in tumor regression (Fig. 4B).

In recent years, there has been a surge in the number of new potential *i*NKT agonists with several structural variations of the prototypical structure of α‐GalCer 1, 2, 3, 4, 5, 6, 7, 8, 9, 10, 11, 12, 13, 14, 15. It has recently been noted that the affinity of TCR binding to CD1d/lipid complexes is not always a predictive parameter of the biological activity of the different *i*NKT‐cell agonists 13, 41, as several parameters, in addition to the TCR‐binding affinity, can modulate the potency of different *i*NKT‐cell agonists in vivo. Our results are in agreement with these conclusions, as we have shown that in the face of in vitro BIAcore measurements, which showed a similar affinity of *i*NKT‐cell TCR binding to mouse CD1d molecules loaded with either ThrCer 6 or α‐GalCer, ThrCer 6 induced higher levels of IFN‐γ secretion in vivo (Fig. 2E) and elicited higher frequencies of antigen‐specific T‐cell responses than did α‐GalCer (Fig. 5). While further experiments are warranted to understand the mechanisms controlling the above results, it is tempting to speculate that the enhanced bioavailability of ThrCer 6 may account for its enhanced potency in vivo. This was particularly noticeable when lower doses of ThrCer 6 were used (Fig. 6A). It has been suggested that the nature of the polar headgroup can determine its recognition by cellular enzymes that may either catabolize some *i*NKT‐cell agonists or redirect them away from the lipid‐antigen presentation pathway 15. Whatever the underlying mechanism, the differences in bioavailability of the different compounds have obvious implications in the clinical settings and will be the subject of further studies.

In conclusion, we have characterized two potent ThrCer‐based analogs, ThrCer 6 and ThrCer 7, in murine and human studies. In in vivo experiments, ThrCer 6 provides robust *i*NKT‐cell stimulation, potent antitumor activity, and prolonged bioavailability as compared to α‐GalCer. These results strongly support the use of ThrCer 6 in phase‐I clinical trials to assess its potency as an immune enhancer to elicit stronger antigen‐specific immune responses.

## Materials and methods

### Mice

C57BL/6 *wild‐type* and C57BL/6 CD1d^–/–^ (NKT‐deficient mice; provided by L. Van Kaer, Vanderbilt University School of Medicine, USA 42. All mice were sex‐matched and aged between 6 and 8 weeks at the time of the first experimental procedure. All studies were carried out in accordance with Animals (Scientific Procedures) Act 1986, and the University of Oxford Animal Welfare and Ethical review Body (AWERB) under project licence 40/3636

### Soluble iNKT‐cell TCR and CD1d–ligand monomers

Soluble human invariant TCR was generated as previously described 34 where both the Vα24 and Vβ11 chains were individually overexpressed in *Escherichia coli*, purified from the inclusion bodies, then refolded, and purified to generate the TCR heterodimers, according to Boulter et al. 43. The murine TCR was generated according to Pellici et al. as a chimeric molecule (with variable murine regions and human constant regions) 44. The hybrid alpha chains and beta chains were individually expressed in *E. coli* and purified from the inclusion bodies, then refolded as above.

### SPR

SPR experiments were performed with a BIAcore 3000 to measure the affinity and kinetics of *i*NKT‐cell TCR binding to human CD1d–ligand complexes. In brief, approximately 1000 RU of the biotinylated hCD1d–lipid complexes were immobilized onto streptavidin‐coated CM5 sensor chips (BIAcore). Aliquots of purified TCR with increasing concentrations were passed over immobilized hCD1d–lipid complex at a flow rate of 10 μL/min for the equilibrium‐binding experiments or 50 μL/min for the kinetics experiments. The dissociation constants, *K*_d_, were calculated by fitting the data from the equilibrium‐binding experiment to a non‐linear regression saturation binding model (GraphPad Prism 5.0), whereas the *k*_off_ data were estimated from the kinetics experiments by fitting the data with the built‐in models of the BIAeval3.1 software (BIAcore).

### Cytokine ELISA

Concentrations of murine IFN‐γ and IL‐4 in serum were determined using standard sandwich ELISAs using the R4‐6A2 capture Ab for IFN‐γ (eBioscience), and the ID11 capture Ab for IL‐4 (Endogen). Biotinylated XMG1.2 (eBioscience) was used as the detection Ab for IFN‐γ and biotinylated BVD6‐24G2 (eBioscience) was used as the detection Ab for IL‐4. Human IFN‐γ in supernatants was determined using the capture Ab NIB42 (BD Pharmingen) and biotinylated 4S.B3 (BD Pharmingen) as the detection Ab.

### iNKT hybridoma assays

Levels of *i*NKT‐cell agonists, presented by CD1d on the surface of cells, were assessed by the amount of IL‐2 secreted by *i*NKT hybridoma DN32.D3 coincubated with the presenting cells for 17–24 h. For in vitro assays, BM‐derived DC (BMDC) were loaded with *i*NKT‐cell agonists for 6 h. Incubation with the agonists then continued for 3, 6, 9, or 24 h before washing and being left in medium without agonist for the remainder of a total of 24 h. Washed cells were than coincubated with DN32.D3. For in vivo assays, Jα18 knockout mice were injected i.v. with 1 μg of ThrCer 6, α‐GalCer or vehicle and 12 or 64 h later CD11c^+^‐enriched splenocytes were coincubated with DN3.D3. Levels of IL‐2 in the supernatant were determined by ELISA. *i*NKT‐cell hybridoma DN32.D3 was kindly provided by A. Bendelac 45.

### Administration of lipids, antigens, and adjuvants

Lipid compounds were solubilized in 150 mM NaCl and 0.5% Tween 20 (vehicle). All substances were diluted in PBS and administered either i.v., or i.m. Unless stated otherwise, doses used per injection were 400 μg OVA (Sigma) and 100 ng of lipid compounds or vehicle.

### Expansion of human iNKT cells, lipid titrations, and DC maturation assays

Human *i*NKT‐cells were expanded as described previously 34. For lipid titration studies, hCD1d C1R cells were pulsed with lipids overnight and following washes, cocultured with human *i*NKT cells. The presence of IFN‐γ was detected in supernatants by ELISA 12. For DC maturation studies, immature DCs were collected on day 4 and pulsed with lipids and *i*NKT cells 34. DC maturation was determined by the upregulation of CD86 by flow cytometry on a FACSCalibur with CellQuest software (BD) and analyzed with FlowJo software (Tree Star). For in vivo murine APC phenotype analysis the expression of CD86 or CD40 on CD11c^+^ APCs from spleen were determined after 18 h post i.v. of 1 μg lipids on a CyAn cytometer and analyzed with FlowJo software (Tree Star).

### Monitoring OVA‐specific immune responses

Mice were bled on day 7 post immunization of lipids and peripheral blood lymphocytes (PBL) stained with tetrameric H‐2 K^b^/OVA_257–264_ peptide complexes, as previously described 27. A representative tetramer staining is shown in Supporting Information Figure 4.

### Determination of B16 melanoma lung metastases

C57BL/6 mice were challenged i.v. with syngeneic B16F10 melanoma, resuspended in PBS. A total of 5 × 10^5^ cells in 200 μL were administered 3 days after administration of indicated doses of *i*NKT‐cell agonists (100, 1, or 0.1 ng). Two weeks after challenge, mice were killed, lungs removed, imaged and the percentage of lung tumor metastases quantified using Adobe Photoshop®.

### Injection of *i*NKT‐cell agonists in tumor‐bearing mice

Mice (*n* = 4–6) were injected subcutaneously (s.c.) with 1 × 10^6^ EG7 cells (a derivative of the thymoma EL4, expressing the OVA protein). Four days later mice were injected i.v. with 800 μg OVA together with either vehicle or 1 μg of the indicated *i*NKT‐cell agonist. Seven days later mice were bled and the number of H‐2K^b^_257–264_ tetramer^+^ cells was assessed by FACS analysis. The size of the tumor was subsequently measured regularly using calipers and expressed as surface area.

## Statistical analysis

All statistical analyses were performed using GraphPad Prism software version 5.0. Student's *t*‐test with two‐tailed analysis was used to compare the level of significance between data sets.

## Conflict of interest

V.C. is serving as consultant for *iOx Therapeutics*, which has an interest in the development of *i*NKT‐cell targeted therapeutics. All other authors declare no financial or commercial conflict of interest.

**Abbreviations**
α‐GalCer:  α‐galactosylceramide
*i*NKT: invariant natural killer T
ThrCer: threitolceramide
SPR: surface plasmon resonance

## Supporting information

As a service to our authors and readers, this journal provides supporting information supplied by the authors. Such materials are peer reviewed and may be re‐organized for online delivery, but are not copy‐edited or typeset. Technical support issues arising from supporting information (other than missing files) should be addressed to the authors.

**Figure S1. ThrCer 6 and ThrCer 7 do not mature DCs in *i*NKT cell deficient mice**. Mice were immunized i.v. with 1 μg of lipids and splenocytes stained with anti‐CD11c and anti‐CD40 mAb to determine the extent of maturation by the expression of CD40 on gated DCs (CD11c+ cells) using flow cytometry. (n=3/group) Median Fluorescent Intensity=MFI. *Error bars are mean ± SEM*.
Figure S2. IFN‐γ in serum of mice injected intramuscularly (i.m.) with iNKT cell agonists. C57BL/6 mice (n=4) or syngeneic CD1d knockout Mice (n=2) were injected intramuscularly with α‐GalCer, ThrCer 6 or vehicle. 18 hours later blood samples were tested for IFN‐γ using ELISA. As controls, mice (n=2) were injected intravenously with α‐GalCer or ThrCer 6. Error bars are mean ±SEM. one of two experiment is shown *p=0.0114.
**Figure S3. Transactivation of NK cells using non‐glycosidic analogues**. Mice were immunized i.v. with 1 μg of lipids and sacrificed at 12 h, 24 h or 33 h post injection (n=3/group). Splenocytes were assessed by flow cytometry for the transactivation of NK cells (DX5+NK1.1+CD3‐ cells) using (B) the surface activation marker, CD69, or (A) intracellular IFN‐γ staining. Error bars are mean ± SEM. *p < 0.05. Representative of two independent experiments
**Figure S4. Gating stratagy for enumerating H‐2Kb/Ova257‐264 specific T cells**. Data relating to numbers of ovalbumin specific T cells was analysed using the following gating stratagy: From top left to right and then bottom left to right. Gating on sing le cells, live cells, B220 negative cells, CD8 positive cells, and finally enumerating the tetrameric H‐2Kb/Ova257‐264 positive cells as percentage of CD8 positive cells.

PRC
