# Supplementary material for: Non‐glycosidic compounds can stimulate both human and mouse iNKT cells
Source: Eur J Immunol. 2016 Mar 1;46(5):1224–34. doi: 10.1002/eji.201546114 (PMC4913735; doi:10.1002/eji.201546114)
Supplement: Supplementary file 1 — Figure S1. ThrCer 6 and ThrCer 7 do not mature DCs in iNKT cell deficient mice. Mice were immunized i.v. with 1 μg of lipids and splenocytes stained with anti‐CD11c and anti‐CD40 mAb to determine the extent of maturation by the expression of CD40 on gated DCs (CD11c+ cells) using flow cytometry. (n=3/group) Median Fluorescent Intensity=MFI. Error bars are mean ± SEM. Figure S2. IFN‐γ in serum of mice injected intramuscularly (i.m.) with iNKT cell agonists. C57BL/6 mice (n=4) or syngeneic CD1d knockout Mice (n=2) were injected intramuscularly with α‐GalCer, ThrCer 6 or vehicle. 18 hours later blood samples were tested for IFN‐γ using ELISA. As controls, mice (n=2) were injected intravenously with α‐GalCer or ThrCer 6. Error bars are mean ±SEM. one of two experiment is shown *p=0.0114. Figure S3. Transactivation of NK cells using non‐glycosidic analogues. Mice were immunized i.v. with 1 μg of lipids and sacrificed at 12 h, 24 h or 33 h post injection (n=3/group). Splenocytes were assessed by flow cytometry for the transactivation of NK cells (DX5+NK1.1+CD3‐ cells) using (B) the surface activation marker, CD69, or (A) intracellular IFN‐γ staining. Error bars are mean ± SEM. *p < 0.05. Representative of two independent experiments Figure S4. Gating stratagy for enumerating H‐2Kb/Ova257‐264 specific T cells. Data relating to numbers of ovalbumin specific T cells was analysed using the following gating stratagy: From top left to right and then bottom left to right. Gating on sing le cells, live cells, B220 negative cells, CD8 positive cells, and finally enumerating the tetrameric H‐2Kb/Ova257‐264 positive cells as percentage of CD8 positive cells. [file EJI-46-1224-s001.pdf]

# European Journal of Immunology

## Supporting Information
## for
## DOI 10.1002/eji.201546114


John-Paul Jukes, Uzi Gileadi, Hemza Ghadbane, Ting-Fong Yu, Dawn Shepherd, Liam R. Cox, Gurdyal S. Besra and Vincenzo Cerundolo

**Non-glycosidic compounds can stimulate both human and mouse *i*NKT cells**

© 2016 The Authors. *European Journal of Immunology* published by WILEY-VCH Verlag GmbH & Co. KGaA, Weinheim.

# Supporting information Figure 1

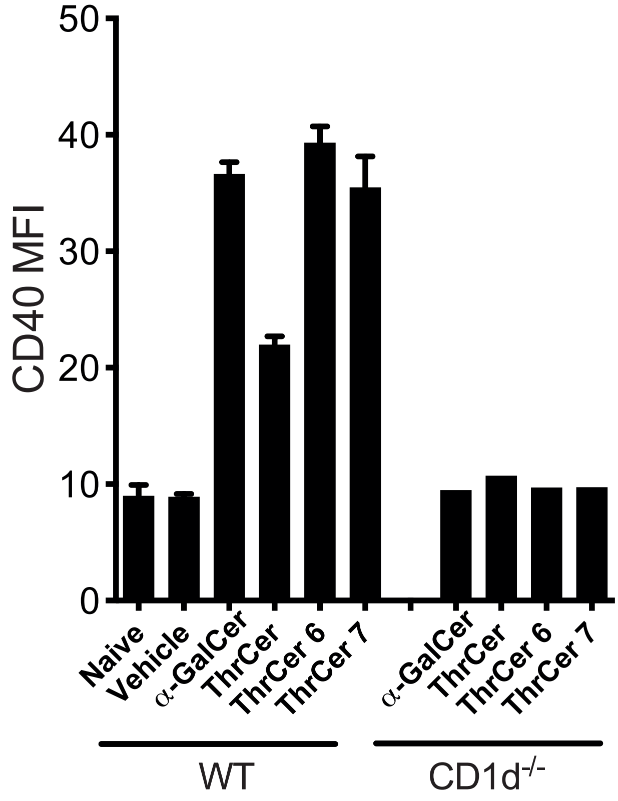


**Figure S1. ThrCer 6 and ThrCer 7 do not mature DCs in *i*NKT cell deficient mice.** Mice were immunized i.v. with 1 μg of lipids and splenocytes stained with anti-CD11c and anti-CD40 mAb to determine the extent of maturation by the expression of CD40 on gated DCs ($CD11c^{+}$ cells) using flow cytometry. (n=3/group) Median Fluorescent Intensity=MFI. *Error bars are mean ± SEM.*

# Supporting information Figure 2

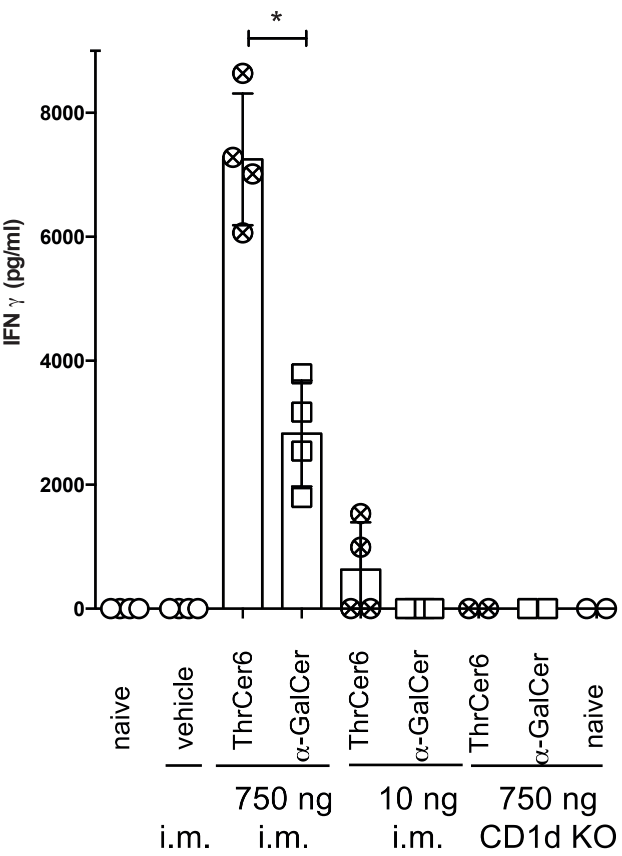


**Figure S2. IFN-γ in serum of mice injected intramuscularly (i.m.) with *i*NKT cell agonists.** C57BL/6 mice (n=4) or syngeneic CD1d knockout Mice (n=2) were injected intramuscularly with α-GalCer, ThrCer 6 or vehicle. 18 hours later blood samples were tested for IFN-γ using ELISA. As controls, mice (n=2) were injected intravenously with α-GalCer or ThrCer 6. *Error bars are mean ±SEM. one of two experiment is shown *p=0.0114.*

# Supporting information Figure 3

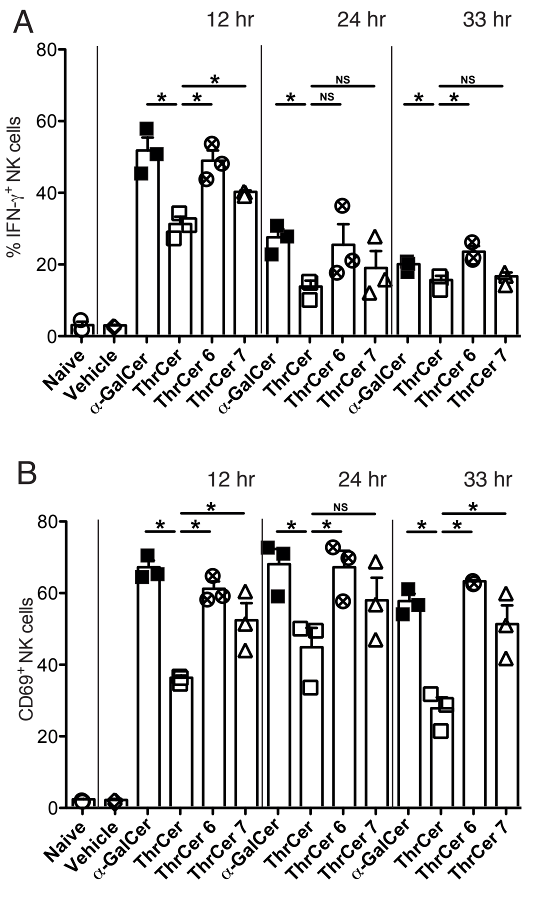


**Figure S3. Transactivation of NK cells using non-glycosidic analogues.** Mice were immunized i.v. with 1 μg of lipids and sacrificed at 12 h, 24 h or 33 h post injection (n=3/group). Splenocytes were assessed by flow cytometry for the transactivation of NK cells (DX5$^{+}$NK1.1$^{+}$CD3$^{-}$ cells) using (B) the surface activation marker, CD69, or (A) intracellular IFN-γ staining. *Error bars are mean ± SEM. *p < 0.05. Representative of two independent experiments*

# Supporting information Figure 4

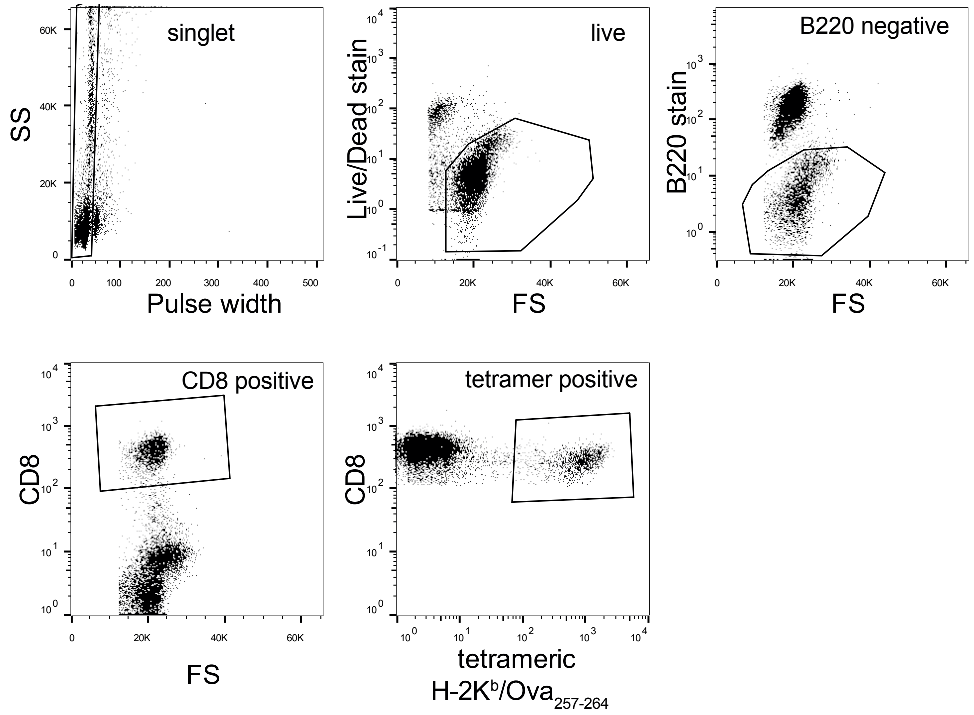


**Figure S4. Gating stratagy for enumerating H-2K$^{b}$/Ova$_{257\text{-}264}$ specific T cells.** Data relating to numbers of ovalbumin specific T cells was analysed using the following gating stratagy: From top left to right and then bottom left to right. Gating on single cells, live cells, B220 negative cells, CD8 positive cells, and finally enumerating the tetrameric H-2K$^{b}$/Ova$_{257\text{-}264}$ positive cells as percentage of CD8 positive cells.
