# Supplementary material for: Non‐glycosidic compounds can stimulate both human and mouse iNKT cells
Source: Eur J Immunol. 2016 Mar 1;46(5):1224–34. doi: 10.1002/eji.201546114 (PMC4913735; doi:10.1002/eji.201546114)
Supplement: Supplementary file 2 — PRC [file EJI-46-1224-s002.pdf]

European Journal of Immunology

Supporting Information
for

DOI 10.1002/eji.201546114

John-Paul Jukes, Uzi Gileadi, Hemza Ghadbane, Ting-Fong Yu, Dawn Shepherd, Liam R. Cox, Gurdyal S. Besra and Vincenzo Cerundolo

Non-glycosidic compounds can stimulate both human and mouse *i*NKT cells

© 2016 The Authors. *European Journal of Immunology* published by WILEY-VCH Verlag GmbH & Co. KGaA, Weinheim.

European Journal of Immunology

Non-glycosidic compounds can stimulate both human and mouse *i*NKT cells

John-Paul Jukes, Uzi Gileadi, Hemza Ghadbane, Ting-Fong Yu, Dawn Shepherd, Liam R. Cox, Gurdyal S. Besra, Vincenzo Cerundolo

Correspondence: Prof. Cerundolo, Vincenzo, Weatherall Institute of Molecular Medicine, Oxford, OX3 9DS, UK

Review Timeline:	Submission date:	08-Oct-2015
	First Editorial decision:	30-Oct-2015
	First Revision received:	07-Dec-2015
	Second Editorial decision:	18-Dec-2015
	Second Revision received:	05-Jan-2016
	Accepted:	08-Feb-2016

Handling Executive Committee member: Prof. Annette Oxenius

Please note that the correspondence below does not include the standard editorial instructions regarding preparation and submission of revised manuscripts, only the scientific revisions requested and addressed.

First Editorial Decision - 30-Oct-2015

Dear Prof. Cerundolo,

Manuscript ID eji.201546114 entitled "Non-glycosidic iNKT cell agonists are potent vaccine adjuvants" which you submitted to the European Journal of Immunology has been reviewed. The comments of the referees are included at the bottom of this letter.

A revised version of your manuscript that takes into account the comments of the referees will be reconsidered for publication. Should you disagree with any of the referees' concerns, you should address this in your point-by-point response and provide solid scientific reasons for why you will not make the requested changes.

You should also pay close attention to the editorial comments included below. *In particular, please edit your figure legends to follow Journal standards as outlined in the editorial comments. Failure to do this will result in delays in the re-review process.*

Please note that submitting a revision of your manuscript does not guarantee eventual acceptance, and that your revision will be re-reviewed by the referees before a decision is rendered.

If the revision of the paper is expected to take more than three months, please inform the editorial office. Revisions taking longer than six months may be assessed by new referee(s) to ensure the relevance and timeliness of the data.

Once again, thank you for submitting your manuscript to European Journal of Immunology and we look forward to receiving your revision.

Yours sincerely,
Karen Chu

On behalf of Prof. Annette Oxenius

Dr. Karen Chu
Editorial Office
European Journal of Immunology
e-mail: ejied@wiley.com
www.eji-journal.eu

****

Reviewer: 1

Comments to the Author
In this paper, Jukes et al. have demonstrated that two ThrCer-based analogs of alpha-galactosylceramide can potently stimulate human iNKT cells and that one of them, ThrCer 6, is able to exhibit more potent anti-tumor activity and prolonged bioavailability than the ancestral compound, alpha-galactosylceramide, in mice. The manuscript is well-written and the study is well-conducted. However, there are two concerns that the authors need to address.

1. Since there is no actual data showing the “adjuvant” effect of non-glycosidic iNKT cell agonists, “vaccine adjuvants” in the title is over statement. In this regard, the word “adjuvant” should be removed from the title and main text to prevent from misleading readers.

2. It seems that the authors have overlooked one key reference, PMC2193764, which showed for the first

time the enhancement of antigen-specific CD8+ T cell response by alpha-galactosylceramide.
Reviewer: 2

Comments to the Author
The development of new iNKT agonist ligands as described in the study of Jukes et al has a clear significance for vaccine development in view of the potent adjuvant activity of iNKT cells. The ThrCer analogs are an improved version of a previously published CD1d ligand, which exhibit higher binding and functional avidity. However, the demonstration of their adjuvant vaccine activity is rather poor and should be further developed.

Major comments:
Figure 3: the antitumor activity of the ThrCer CD1d ligands is shown in prophylactic settings in a tumor model in which antitumor effect is mainly achieved by the iNKT cell-mediated transactivation of NK cells, rather than via an adaptive immune response, which is the scope of the study. In this context the antitumor activity should be tested in peptide vaccine settings.
Figure 4: the adjuvant activity of one of the two ThrCer analogs is shown in a single graph at a single time-point post vaccine. The only parameter is the dose of the analog while it would be important to see the kinetic of T cell expansion, their persistence as well as their effectiveness in antitumor therapeutic settings (remark Fig3). In addition another route than i.v. should be tested, for instance i.m. which would be better accepted for clinical application.
In conclusion, despite the high sensitivity of these analogs, the presented data are not very convincing for new vaccine adjuvant but rather describe two additional aGalCer analogs.

First Revision – authors' response - 07-Dec-2015

Reviewer 1:

We are very pleased that reviewer 1 found our manuscript " well written''…'" and that "the study is well-conducted".

1) Since there is no actual data showing the "adjuvant" effect of non-glycosidic iNKT cell agonists, "vaccine adjuvants" in the title is over statement. In this regard, the word "adjuvant" should be removed from the title and main text to prevent from misleading readers.

To address this criticism we have changed the title of the manuscript and avoided the term "adjuvant" in the text. In addition, we showed two additional experiments (see Figure 4 panel B and Supporting Figure S4), in which we have confirmed and extended the ability of ThrCer 6 to enhance antigen

specific T cell responses and cross-presentation of soluble antigens, which led to the regression of implanted tumors in therapeutic settings. Furthermore, as requested by reviewer 2, we have shown that intra-muscular injection of ThrCer 6 enhances iNKT cells activation, as defined by IFNγ secretion in wild type, but not in CD1d-/- mice (Supporting Figure S2).

2) It seems that the authors have overlooked one key reference, PMC2193764, which showed for the first time the enhancement of antigen-specific CD8+ T cell response by alpha-galactosylceramide.

We apologise for this oversight and have added the suggested reference (reference 21) in the introduction (Gonzalez-Aseguinolaza et al 2002).

Reviewer 2:

We wish to thank reviewer 2 for acknowledging that “the development of new iNKT agonist ligands as described in the study of Jukes et al has a clear significance for vaccine development in view of the potent adjuvant activity of iNKT cells.”

Major comments:

1) Figure 3: the antitumor activity of the ThrCer CD1d ligands is shown in prophylactic settings in a tumor model in which antitumor effect is mainly achieved by the iNKT cell-mediated transactivation of NK cells, rather than via an adaptive immune response, which is the scope of the study. In this context the antitumor activity should be tested in peptide vaccine settings.

We agree with the reviewer’s comment and addressed his/her comment by adding the results describing the ability of ThrCer 6 in combination with soluble Ovalbumin to induce regression of Ovalbumin expressing tumors in a therapeutic setting (Supporting Figure S4).

2) Figure 4: the adjuvant activity of one of the two ThrCer analogs is shown in a single graph at a single time-point post vaccine. The only parameter is the dose of the analog while it would be important to see the kinetic of T cell expansion, their persistence as well as their effectiveness in antitumor therapeutic settings (remark Fig3). In addition another route than i.v. should be tested, for instance i.m. which would be better accepted for clinical application.

To address the reviewer’s comment on the kinetics of the immune response elicited by low concentrations of ThrCer 6, we added another panel to Figure 4 (Figure 4B), which shows the kinetic of

European Journal of Immunology

the frequency of H-2Kb/OVA257–264 tetramer+ CD8+ T cells on days 7 and 12 in the blood of mice injected with two doses (10 ng and 750 ng) of either ThrCer 6 or α-GalCer. The results of these experiments indicate that the adjuvant effect of ThrCer 6 persists for 12 days after the injection and that the frequency of H-2 Kb/OVA257–264 tetramer + CD8+ T cells continues to be higher at day 12 than in mice injected with α-GalCer. This difference reaches statistical significance in mice injected with lower doses of ThrCer 6 and α-GalCer.

In addition, we have performed additional experiments to assess whether another route of injection of ThrCer6 results in iNKT cell activation. The results of these experiments demonstrated that i.m. injection of ThrCer 6 results in rapid iNKT cell activation, as defined by IFNγ secretion within 18 hours from injection, to levels which were even higher than those observed in mice injected with α-GalCer.
We confirmed that this effect was iNKT cell dependent by demonstrating lack of IFN γ secretion in CD1d-/- mice.

We are convinced that the additional experiments that we have performed have significantly strengthened the significance of our findings and we hope that the editor and the referees will find the revised manuscript acceptable for publication.

Second Editorial Decision 18-Dec-2015

Dear Prof. Cerundolo,

Thank you for submitting your revised manuscript ID eji.201546114.R1 entitled "Ability of non-glycosidic compounds to stimulate human and mouse <i>i</i>NKT cells" to the European Journal of Immunology. Your manuscript has been re-reviewed and the comments of the referee are included at the bottom of this letter.

The referee has recommended publication, with a very minor revision to your paper. Therefore, I invite you to respond to the comments of the referees and revise your manuscript accordingly.

You should also pay close attention to the editorial comments included below. *In particular, please edit your figure legends to follow Journal standards as outlined in the editorial comments. Failure to do this will result in delays in the re-review process.*

Once again, thank you for submitting your manuscript to European Journal of Immunology. We look forward to receiving your revision.

Yours sincerely,
Karen Chu

on behalf of Prof. Annette Oxenius

Dr. Karen Chu
Editorial Office
European Journal of Immunology
e-mail: ejied@wiley.com
www.eji-journal.eu
****

Reviewer: 2

Comments to the Author

I have appreciated the revision you have made and the additional results/text changes you have added, have improved significantly the manuscript. I only regret that the new results of tumor growth kinetic and frequency of Kb/OVA CTLs were included as a supporting Fig.4 instead of being merged or even replacing the actual Fig.4. Could you consider that?

<u>Second Revision – authors' response - 05-Jan-2016</u>

We wish to thank the reviewers and the editors for their constructive comments on our manuscript "Ability of non-glycosidic compounds to stimulate human and mouse iNKT cells". We have agreed with all the changes suggested by the editors and the additional suggestion made by the reviewer and have amended the text accordingly (see text highlighted in red),

Reviewer 2 had made the point that he ".. appreciated the revision you have made and the additional results/text changes you have added, have improved significantly the manuscript. I only regret that the new results of tumor growth kinetic and frequency of Kb/OVA CTLs were included as a supporting Fig.4 instead of being merged or even replacing the actual Fig.4. Could you consider that?"

We have therefore addressed this suggestion by relocating the old supporting figure 4 into the main figures as new figure 4. Subsequent figures and the main text have been renumbered and amended accordingly.

We are satisfied that the additional changes improve the manuscript and we hope that the editor and the referees will find the revised manuscript acceptable for publication.

Third Editorial Decision - 15-Jan-2016

Dear Prof. Cerundolo,

It is a pleasure to provisionally accept your manuscript entitled "Non-glycosidic compounds can stimulate both human and mouse iNKT cells" for publication in the European Journal of Immunology.

For final acceptance, please follow the instructions below and return the requested items as soon as possible as we cannot process your manuscript further until all items listed below are dealt with.

Please note that EJI articles are now published online a few days after final acceptance (see Accepted Articles: http://onlinelibrary.wiley.com/journal/10.1002/(ISSN)1521-4141/accepted). The files used for the Accepted Articles are the final files and information supplied by you in Manuscript Central. You should therefore check that all the information (including author names) is correct as changes will NOT be permitted until the proofs stage.

We look forward to hearing from you and thank you for submitting your manuscript to the European Journal of Immunology.

Yours sincerely,
Laura Soto Vazquez

on behalf of Prof. Annette Oxenius

Editorial Office
European Journal of Immunology
e-mail: ejied@wiley.com
www.eji-journal.eu
